# Case report: Diagnostic challenge: a new multiple sclerosis “relapse” leading to the diagnosis of anaplastic astrocytoma

**DOI:** 10.3389/fneur.2023.1324269

**Published:** 2024-01-26

**Authors:** Martina Petrášová, Iva Šrotová, Jan Kolčava, Pavel Štourač, Ludmila Hynková, Miloš Keřkovský, Hana Pikulová, Eduard Neuman, Leoš Kren, Eva Vlčková

**Affiliations:** ^1^Faculty of Medicine, Masaryk University Brno, Brno, Czechia; ^2^Department of Neurology, University Hospital Brno, Brno, Czechia; ^3^Department of Radiology, Masaryk Memorial Cancer Institute, Brno, Czechia; ^4^Department of Radiology and Nuclear Medicine, University Hospital Brno, Brno, Czechia; ^5^Department of Neurosurgery, University Hospital Brno, Brno, Czechia; ^6^Department of Pathology, University Hospital Brno, Brno, Czechia

**Keywords:** astrocytoma, multiple sclerosis, magnetic resonance imaging, positron-emission tomography, case report

## Abstract

Cerebral tumors and multiple sclerosis (MS) can show overlapping clinical and magnetic resonance imaging (MRI) features and even occur concurrently. Due to the emergence of new symptoms, not usually MS related, an MRI was conducted in a 29-year-old woman with relapsing-remitting MS and showed a significant size progression of a parieto-occipital lesion, with mild clinical correlates, such as blurred vision, difficulty in speaking, and headache. Contrast-enhanced MRI and fluorothymidine positron-emission tomography (PET) did not point toward neoplasm, a lesion biopsy, however, showed astrocytoma, which was confirmed as grade III astrocytoma after the radical resection of the tumor. In the case of an atypical lesion, a tumor should be considered in patients with MS. A small fraction of high-grade gliomas show no enhancement on MRI and no hypermetabolism on PET. Biopsy proved to be the essential step in a successful diagnostic workup. To the best of our knowledge, this is the first case of anaplastic astrocytoma with these radiological features reported in a patient with MS.

## 1 Introduction

Multiple sclerosis (MS) is a chronic inflammatory disease of the central nervous system (1). MS lesions can vary in size from millimeters to one or two centimeters in diameter (2). Lesions are only larger than 2 cm in a rare tumefactive variant of MS. This variant may radiographically closely resemble a brain tumor and present itself clinically with symptoms generally associated more frequently with tumors (3).

Primary brain tumors are a rare type of cancer in adults. Two thirds of these come from astrocytic cell lineage. Tumors can present in patients as headaches, focal deficits—cognitive, motor, sensory or linguistic, seizures or visual problems (4), all depending on the location of the lesion. The incidence of primary brain tumors in the MS patient population is estimated at 0.27% (5). The risk of metastatic tumorous lesions in the brain can differ in some cases in the MS population compared to the general population. However, the reports stating higher or lower incidences of specific tumor types are not completely consistent. Lung, breast and kidney cancer and melanoma are most commonly associated with brain metastases (6). Of these types, urinary system cancer was repeatedly found to be increased in the MS population, however, surveillance bias cannot be excluded (5). The incidence of brain metastases in the general population ranges from 7 to 14/100,000 people per year (7).

MRI constitutes a standard in neuroimaging of brain tumors (8). In cases where MRI is not decisive and suspicion of neoplasm is high, we may consider applying other modalities, such as PET.

Case reports of gliomas coexisting with multiple sclerosis (MS) have been described in the literature since 1973 (9). Due to the complex clinical and radiological traits inherent to both entities, this concurrent phenomenon remains challenging to diagnose.

We present a case report of a female MS patient with anaplastic astrocytoma, that had an appearance of tumefactive lesion on MRI. Adequate clinical correlation was missing. Fluorothymidine-PET showed no accumulation of the radiopharmaceutical. The case report below is written with respect to the CARE checklist.

## 2 Case report

The patient experienced the initial symptoms of MS (optic neuritis on the left eye followed by paresthesias of the right hand) at the age of 17. She fulfilled the diagnostic criteria of MS (10) (juxtacortical and periventricular demyelination lesions hyperintense in T2-weighted and Fluid Inversion Recovery sequences (a small lesion was also visible in the location of the future tumor), spinal cord lesions hyperintense in T2/Turbo Inversion Recovery Magnitude sequence, and 12 oligoclonal bands in the cerebrospinal fluid; the results of cerebrospinal fluid analysis were otherwise unremarkable: the protein level was 0.37 g/L and there were no cells found in the analysis performed at the time of initial diagnosis). Therefore, the patient was treated with high-dose steroids (3 g of intravenous methylprednisolone). Her clinical symptoms and signs completely resolved in 2 weeks, and subsequently, the long-term treatment with glatiramer acetate (40 mg/three times a week) as disease-modifying therapy (DMT) was started. At the age of 26, the DMT escalation to the dimethyl fumarate (240 mg/twice a day) was performed because of clinical progression.

The details of particular symptoms/signs, brain magnetic resonance imaging (MRI) changes, DMT as well as the treatment of particular attacks and the Expanded Disability Status Scale (EDSS) at the most important time points of the patient's clinical course are described in Table 1.

**Table 1:** Clinical course, treatment and brain MRI changes at the most important time points of the patient's disease.

	**Patient's age**	**Newly developed clinical symptoms/signs**	**Treatment of acute relapse (intravenous methylprednisolone)**	**Brain MRI**	**EDSS at the maximum of clinical symptoms**	**Resolution of symptoms**	**DMT**
2009	17	Optic neuritis on the left eye and positive sensory symptoms of upper limb	1 g daily for 3 days	Demyelinating lesions in supra- and infratentorial region, hyperintense in T2-weighted and Fluid Attenuated Inversion Recovery sequences	1.5	Complete	GA
2015	23	Mild paraparesis, negative sensory symptoms of the right upper limb and right lower limb	1 g daily for 3 days	Radiological activity—postcontrast (gadolinium) enhancement in three supratentorial lesions, and progression in lesion number	2.0	Complete	GA^*^
2016	24	Vertigo and mild urinary retention	1 g daily for 3 days	No significant changes	3.0	Incomplete	GA^*^
2018	26	Mild spastic paraparesis, more expressed on the right lower limb	1 g daily for 3 days	No significant changes	3.5	Complete	GA^*^
2019	27	Progression of vertigo, very mild dysarthria	1 g daily for 3 days	No significant changes	2.0	Incomplete	DMF^**^
2021	29	Dull occipital headache, blurred vision in the right half of the visual field, mild anomic aphasia, fatigue	1 g daily for 5 days	Size progression of the left parieto-occipital corticosubcortical lesion, which had an expansive character and was hyperintense in T2-weighted sequence and hypointense in T1-weighted sequence and showed no contrast enhancement	3.5	Incomplete	DMF

GA, glatiramer acetate; DMF, dimethyl fumarate; EDSS, expanded disability status scale.

^*^Despite mild clinical and radiological progression, the patient did not fulfill the local criteria for DMT escalation (at the appropriate time point, the escalation to 2nd line treatments was approved only in patients with two relapses in 1 year with parallel progression on the MRI).

^**^Due to the change of reimbursement criteria of the local health insurance companies, the patient's medication was escalated to DMF.

**Figure 1:**
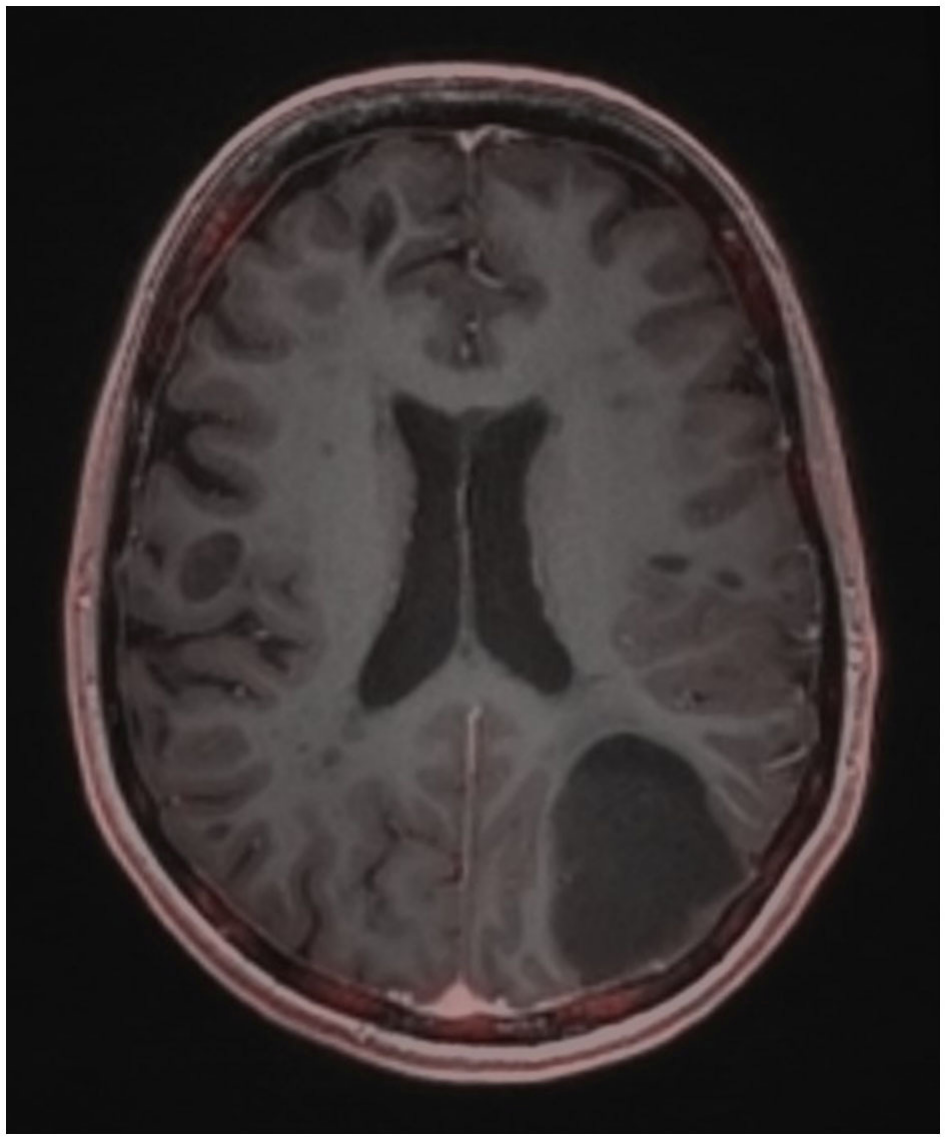
Brain MRI using contrast agents.

At the age of 29, clinical symptoms of blurred vision mainly in the right half of the visual field, mild anomic aphasia, extreme fatigue, and dull headache bilaterally in the occipital region occurred. The patient reported that the pain was a little more intense early in the morning after waking up. These symptoms developed slowly over 2 months. MRI of the brain revealed size progression of the left parieto-occipital cortico-subcortical lesion (42 × 42 × 34 mm vs. 32 × 30 × 25 mm in the previous MRI), which had an expansive character and showed no gadolinium enhancement (Figure 1). Moreover, the lesion was described by the radiologist as heterogenous, hyperintense in T2-weighted and Fluid Attenuated Inversion Recovery sequences, hypointense in T1-weighted sequence and there was no restriction of diffusion observed. With regard to the size and character of the lesion, the anomic aphasia could be explained by edema spreading to the angular gyrus. The patient reported blurred vision bilaterally (predominantly in the right visual field) and this symptom, according to the ophthalmologist, was most probably associated with Uhthoff's phenomenon.

**Figure 2:**
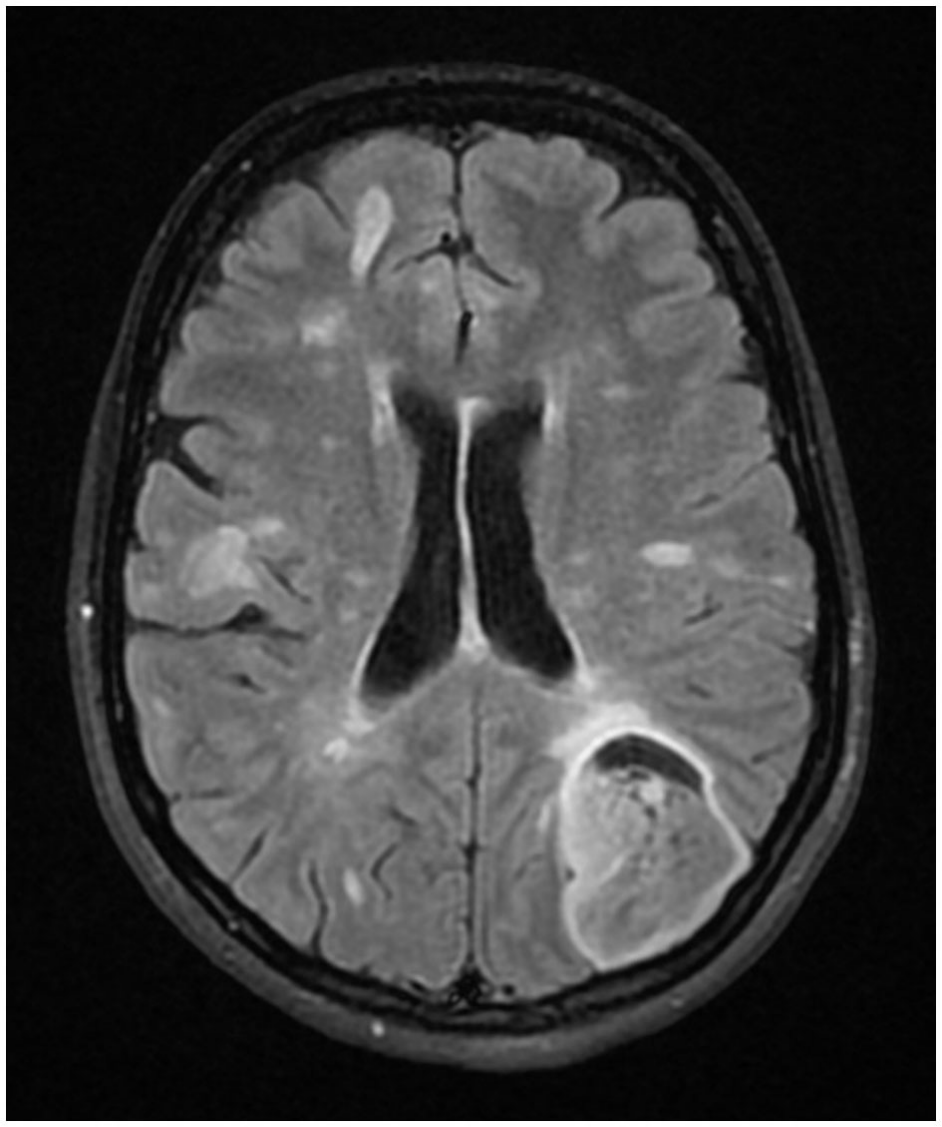
18F-fluorothymidine PET-MRI of the brain.

In the context of MRI progression and newly developed clinical symptoms, the patient was treated with 5 g of methylprednisolone. The follow-up MRI showed no progression of the lesion; however, the symptoms showed no significant improvement after steroid treatment with the exception of a transitory reduction of the headache intensity. The non-MS etiology of the lesion was thus considered. ^18^F-fluorothymidine PET-MRI did not demonstrate an increased accumulation of radiopharmaceutical within the lesion and there was no post-contrast enhancement of the lesion (Figure 2). A tumefactive lesion was still considered probable on the basis of this examination. This finding, however, cannot definitely exclude the tumor diagnosis for several reasons mentioned in the discussion below.

Because of the clinical progression, DMT escalation was considered. The biopsy of the lesion was performed to definitely exclude the tumor as the etiology of the patient's clinical status before the DMT escalation. The histological examination of the biopsy sample revealed anaplastic astrocytoma.

The craniectomy and radical extirpation of the tumor was performed. The histological examination confirmed the diagnosis of anaplastic astrocytoma grade III with mutation in isocitrate dehydrogenase.

The prognosis of this type of tumor is rather poor, with a mean 5-year survival of 28.6% (11), which increases to 41.8% if the patient receives adjuvant chemo- and radiotherapy (12). For overall survival, positive prognostic factors have been described: age (<41), isocitrate dehydrogenase mutation, radical resection and Karnofsky performance status of at least 90% (13), however, another source states a positive effect of Karnofsky performance status of at least 70% (14). In the latter case (Karnofsky performance status of 70%), our patient would have all of the positive factors mentioned above.

A neuropsychological examination was performed pre- and postoperatively and this included a battery of tests (Wechsler Adult Intelligence Test—Symbol Encoding Test and Digit Span Forward and Backward Test, Trail Making Test A/B, Stroop Task, Verbal Fluency Test, Rey-Osterrieth complex figure and Montreal Cognitive Assessment). The patient showed improvement in several tests (tests of attention and psychomotor processing speed, working memory, visual-constructive abilities and Montreal Cognitive Assessment) after surgery. Tests for executive function remained unchanged.

The patient started fractionated radiotherapy with a total dose of 59.4 Gy (33 × 1.8 Gy), followed by chemotherapy (temozolomide, 12 cycles in total/28-day cycle: 150 mg/m^2^ of body surface (i.e., 220 mg) once a day for five consecutive days). This therapy follows the guidelines for this type of tumor (15). She also discontinued the dimethyl fumarate medication because of the cumulative risk of leukopenia, to which the patient was prone (last leukocyte count before chemotherapy was below the lower limit of normal range: 3.080 × 109/l), and started with the administration of glatiramer acetate (40 mg/three times a week), which is allowed for patients with MS and active oncological disease with anti-cancer treatment.

Currently (at the age of 30), the patient has been clinically and radiologically stable for 18 months and presents with several persisting symptoms, including combined speech deficit with mild dysarthria and very mild anomic aphasia, and blurred vision emerging after physical activity (which was attributed to Uhthoff's phenomenon). The Karnofsky performance status is 70 due to inability to work (16), current EDSS score is 3 (functional systems: ambulation 0, pyramidal 2, cerebellar 0, brainstem 2, sensory 1, visual 1, cerebral 2, bowel and bladder 0).

The episodes of care are summarized in Table 2.

**Table 2:** Timeline of the episodes of care.

**Month/year**	**New clinical symptoms/examination/intervention**	**Test or treatment results**	**Notes**
03/2021	Blurred vision, headache, anomic aphasia, fatigue	Clinical progression (aphasia, blurred vision, new type of headache) leading to MRI examination and treatment of the presumed relapse (see next two lines)	
03/2021	MRI	Size progression of the left parieto-occipital corticosubcortical lesion	Tumefactive appearance
03/2021	Treatment of presumed acute relapse of MS: methylprednisolone IV, 1 g daily for 5 days	Only a minimal effect on headache and no impact on other clinical symptoms	
04/2021	Follow-up MRI	No significant change in the lesion	
06/2021	PET/MRI	No accumulation of the radiopharmaceutical within the lesion	Tumefactive appearance of the lesion
07/2021	Planned DMT escalation (blood tests, chest X-ray, exclusion of focus of infection)	No contraindication found	Given the remaining uncertainties regarding the tumefactive lesion, lesion biopsy was planned prior to DMT escalation
09/2021	Stereotactic biopsy	Anaplastic astrocytoma	A tumor resection was planned
10/2021	Radical resection of the tumor	Anaplastic astrocytoma grade III confirmed	Based on the results of histological examination of the tissue taken during resection, chemo- and radiotherapy was planned
11/2021	Fractionated radiotherapy	59.4 Gy (33 × 1.8 Gy)	
03/2022	Adjuvant chemotherapy: temozolomide: 12 cycles in total/28-day cycle: 150 mg/m^2^ of body surface daily for five consecutive days	Clinically stable with very mild headache, blurred vision following physical activity; MRI with no residual tumor detected	

MRI, magnetic resonance imaging; MS, multiple sclerosis; IV, intravenously; PET/MRI, positron emission tomography/magnetic resonance imaging; DMT, disease-modifying treatment.

## 3 Discussion

To the authors' knowledge, this is the first published report of anaplastic astrocytoma non-accumulating on fluorothymidine PET imaging and non-enhancing on MRI in an MS patient.

In the case of atypical MS plaques, the issue of possible concurrence of MS and brain tumor may arise. Several clinical and radiological aspects should be considered in this situation and the biopsy of the lesion may in some cases be needed for final resolution. Clinically, any uncommon neurological symptoms and signs in MS patients should suggest more extensive investigations to exclude overlapping pathologies (17). The same applies for slowly developing symptoms with no spontaneous remission and no improvement following acute relapse medication. All these atypical features suggested non-MS etiology of the lesion in our patient.

Neuroimaging mostly helps to resolve the situation. Tumefactive demyelinating lesions are generally defined as acute, large (>2 cm) lesions. On imaging, they usually present with relatively little mass effect or surrounding edema, contrast enhancement in an open-ring pattern, high ADC values, and low relative cerebral blood volume (18–20). While the great majority of tumefactive demyelinating lesions and brain tumors enhance with gadolinium contrast, some of the lesions of both types may show no gadolinium enhancement, which was the case with our patient.

The use of conventional fluorodeoxyglucose PET in the detection of brain tumors is limited as it constitutes a high background signal (21). If the primary brain tumor, especially grade IV glioma, is considered, fluorothymidine PET imaging provides higher detection sensitivity. However, the detection rate decreases with the lower grade gliomas (22). Moreover, a correlation between fluorothymidine uptake and contrast enhancement on MRI in high-grade gliomas has been reported (23) since the fluorothymidine concentration in the tissue depends strongly on the disruption of the blood-brain barrier (24).

The stereotactic biopsy of the lesion and its histologic examination represents the final diagnostic approach in equivocal cases (25). It is safe and reliable, especially if specimens from multiple sites within the lesion are targeted. According to published data, the diagnostic accuracy of the stereotactic biopsy reaches 82%−99% (26).

However, as in every case report, the ability to generalize particular findings mentioned in our case report to the broader patient population is limited.

## 4 Patient perspective

The interdisciplinary team consisting of neurology, neurosurgery, oncology and psychiatry specialists provides the patient with consistent support. Subjectively, the patient experiences only subtle neurological disability. Ongoing fatigue does, however, preclude her from keeping up with her occupation. Her mood is stabilized and motivation to continue with the therapy is high.

“Radiotherapy and chemotherapy were manageable. The only thing that bothers me is the daily headache, although it is mostly mild. And fatigue, which makes me unable to do everything as before. But I'm glad I'm self-sufficient.”

## 5 Conclusion

Tumefactive lesions with atypical clinical symptoms not responding to the acute corticosteroid medication should raise a “red flag” of non-MS etiology. ^18^Fluorothymidine PET is frequently used to confirm or exclude the tumor, but its sensitivity in lower-grade gliomas might be limited. The stereotactic biopsy of the lesion represents the most important method in cases without clinical-radiological correlation. The possibility of dual pathology must always be kept in mind.

## Data availability statement

The original contributions presented in the study are included in the article/supplementary material, further inquiries can be directed to the corresponding author.

## Ethics statement

Written informed consent was obtained from the individual(s) for the publication of any potentially identifiable images or data included in this article.

## Author contributions

MP: Conceptualization, Writing—original draft. IŠ: Conceptualization, Methodology, Supervision, Writing—review & editing. JK: Supervision, Writing—review & editing. PŠ: Supervision, Writing—review & editing. LH: Supervision, Writing—review & editing. MK: Supervision, Writing—review & editing. HP: Supervision, Writing—review & editing. EN: Supervision, Writing—review & editing. LK: Supervision, Writing—review & editing. EV: Supervision, Writing—review & editing.
